# Modification of Laser Marking Ability and Properties of Polypropylene Using Silica Waste as a Filler

**DOI:** 10.3390/ma14226961

**Published:** 2021-11-17

**Authors:** Artur Kościuszko, Piotr Czyżewski, Mateusz Rojewski

**Affiliations:** Department of Manufacturing Techniques, Faculty of Mechanical Engineering, Bydgoszcz University of Science and Technology, Al. Prof. S. Kaliskiego 7, 85-796 Bydgoszcz, Poland; artur.kosciuszko@pbs.edu.pl (A.K.); piotr.czyzewski@pbs.edu.pl (P.C.)

**Keywords:** silica waste, polypropylene, laser marking, mechanical properties, heterogeneous nucleation

## Abstract

Polypropylene (PP) belongs to the group of polymers characterized by low susceptibility to absorption of electromagnetic radiation in the infrared range (*λ* = 1064 nm). This research consisted of assessing the possibility of using silica waste from the metallurgic industry as an additive for PP laser marking. The modifier was introduced into the polymer matrix in the range from 1 to 10 wt%. The effects of laser radiation were assessed based on colorimetric tests and microscopic surface analysis. The mechanical properties of the composites were determined during the static tensile tests. The thermal properties were investigated via differential scanning calorimetry. It was found that the introduction of silica waste into polypropylene allows for the effective marking of sample surfaces with the use of a laser beam. The greatest contrast between the graphic symbol and the background was obtained for silica contents of 3 and 5 wt%, with the use of a low-speed laser head and a strong concentration of the laser beam. The application of silica caused an increase in the modulus of elasticity and the tensile strength of the composite samples. Increases in the crystallization temperature and the degree of crystallinity of the polymer matrix were also observed. It was found that silica waste can act as multifunctional additive for polypropylene.

## 1. Introduction

Polypropylene (PP) is one of the most commonly used thermoplastic polymers in industry. The demand for this material accounts for approximately 19% of the total global procurement of polymeric materials, due to its relatively good physical properties, easy processing, and low price. Moreover, the widespread use of PP in various branches of industry results from the possibility of easy modification of its mechanical and thermal properties. For ecological reasons, raw materials of natural origin are increasingly used as PP fillers. These include, among others, ground wood particles [1,2,3], natural fibers [4], and waste materials, e.g., sunflower husks [5], crushed egg shells [6], and ground rubber from car tires [7,8]. Our previous research [9] describes the mechanical properties of polypropylene composites with 10 and 20 wt% silica content.

Silica—as with talc, kaolin, metal oxides, and carbonates—belongs to the group of inorganic powder fillers. The morphology of the filler particles (dimensions and surface) influences the mechanical properties of the composites [10]. It is assumed that the stiffness of composite materials is highly dependent on their filler content. Increasing the content of particles in the polymer matrix usually results in an increase in the value of Young’s modulus, and this relationship is usually nonlinear. Inorganic powder fillers are usually spherical or lobed in shape, but if the reinforcement effect is desired, the best results are obtained when the silicates have a lamellar shape. Research confirms that when spherical particles are used, the tensile strength of composites decreases from a certain threshold value with increasing filler content [11]. Nevertheless, Pukanszky [12] indicates that using particles with a suitably small diameter can make it possible to obtain a reinforcement effect. Along with the decrease in the size of the filler particles and the increase in the specific surface area, an increase in the strength of the composite is observed, while the changes are combined with decreases in elongation and impact strength [13]. As a result of introducing ~5 wt% nanosilica to PP, Rong [14] achieved an increase of less than 20% in the tensile strength of the composite, compared to the unfilled polymer.

The introduction of powder fillers to the thermoplastic matrix, in addition to enhancing mechanical properties, usually improves processing properties (shrinkage reduction), and increases thermal stability and thermal conductivity [15,16]. In addition, the consumption of plastics is reduced, which in some cases reduces the cost of the material. On the other hand, a specific effect of introducing powder fillers to PP is the modification of the crystallization temperature of the polymeric material [17]. The filler particles may act as heterogeneous nuclei of crystallization, resulting in the initiation of the crystallization at higher temperatures [18]. In the research conducted by Garcia [19], the introduction of silica powder into PP resulted in an increase in the crystallization temperature from 117 to 121 °C. It is known that this effect allows the shortening of the cooling time of PP moldings in the mold cavity. Another effect of introducing powdered inorganic fillers into the PP matrix may be an increase in the susceptibility of the material to laser light, which allows graphic signs to be applied to the surface of products made from polymer materials [20].

The use of laser beams is progressively becoming a common method of modifying the surface layers of materials. Due to the possibility of obtaining perfect monochromaticity, strong laser beam concentration, and directivity, laser light has become the subject of several experimental studies in recent years [21,22,23,24]. A strongly developed tendency of applications for this type of electromagnetic radiation is laser marking. This method allows graphic signs to be obtained in the form of letters, numbers, symbols, barcodes, or drawings on the surfaces of various materials, including metals [25], wood [26], ceramics [27], and plastics [28]. Laser marking makes it possible to obtain graphic signs that are resistant to abrasion, water, and air humidity [29,30]; in addition, it has a positive effect on the environment, reducing the amount of material used in conventional decoration methods such as printing, padding, painting, and in-mold labeling (IML) [31]. Laser marking is a non-contact process; concentrated high-frequency laser pulses change the material properties in a specific surface area. The effect of this is the initiation of internal stresses, which result in cracking and deformation of the surface layer elements. The color change occurs most often as a result of physicochemical processes taking place on the marked surface, such as ablation, foaming, charring, and discoloration [32,33]. The assumed geometrical features of a graphic sign (size, shape, brightness, etc.) can be derived by using appropriate parameters of the laser beam (i.e., lens focal length, wavelength, duration, and energy of laser pulses) [34]. Low cost and the ability to mark a variety of surfaces make that the most commonly used industrial lasers are those that use energy from the near-infrared range, with a wavelength of 1064 nm. Due to the variety of properties of plastics, marking them is in many cases difficult or completely impossible. Easy carbonization and high carbon content make materials such as polycarbonate [35] and polystyrene [36] highly susceptible to marking with the use of radiation at the wave length λ = 1064 nm. Because of low light absorption at the same wavelength, marking elements made of polyethylene [37] and polypropylene [38] requires the use of laser marking additives (LMAs).

Additives to laser marking are mainly divided into inorganic, organic, and polymer/inorganic composite materials. Recently, there has been an increase in research and implementation projects in which the use of inexpensive or direct waste compounds as additives is examined. In such cases, powdered metal oxides such as Fe_3_O_4_ [39], Sb_2_O_3_ [40], or Bi_2_O_3_ [41] are most often used. Zheng Cao et al. [42] showed that 0.02% MoS_2_ content and the selection of appropriate marking parameters result in obtaining graphic signs with high contrast. Wen et al. [43] found that the addition of only 0.005 wt% graphene in the matrix of the material acts as an effective absorber of laser pulses in the near-infrared range. Zhou et al. [44] proved that the use of carbon nanotubes affects the obtaining of high contrast between the graphic symbol and the background in the case of PP marking. The addition of inorganic compounds to polymers is a simple and easy method to solve problems arising from the insensitivity of some materials to near-infrared lasers [45].

As described above, researchers have conducted investigations related to increasing the laser marking ability of polymers using inorganic chemical compounds. There are also publications describing the influence of micro- and nanosilica on the thermal and mechanical properties of polypropylene composites. The testing of functional properties is important for the evaluation of the application potential of the developed LMA modifier. However, no publications describing the relationship between the use and the content of silica (especially silica waste) in the PP matrix on the one hand, and the effectiveness of laser marking for injection moldings on the other, have been published to date. The described experiment can confirm the possibility of using a single additive (in this case, of waste origin), which may have multiple functions (filling, dyeing, and the possibility of laser marking). For economic, technological, and environmental reasons, it is advantageous to look for similar solutions for industrial implementation.

The aim of the research was to assess the impact of different waste silica contents (from 1 to 10 wt%) on the effectiveness of laser marking of polypropylene samples. Moreover, in order to assess the possibility of applying the modifier in practical situations, tests of processing and functional properties were carried out. The relationships between the silica content in the material and the crystallization temperature, degree of crystallinity, density, and mechanical properties were determined.

## 2. Materials and Methods

### 2.1. Materials

Moplen HP500N commercial polypropylene (Basell Orlen Polyolefins, Płock, Poland) was used in our research. This polymer is intended for processing by injection molding. The value of the mass-flow rate (*MFR*) was 12 g/10 min (230 °C/2.16 kg), the tensile modulus (*E*) was 1400 MPa, and the tensile stress at yield (*σ_y_*) was 35 MPa [46].

**Figure 1:**
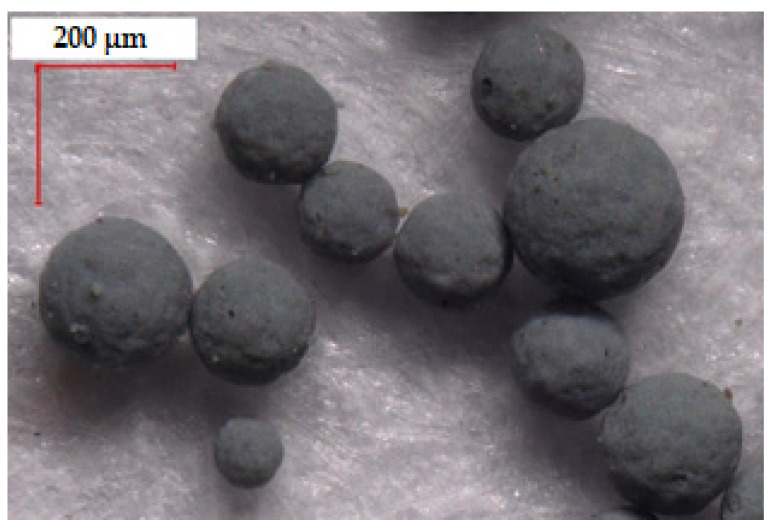
Silica agglomerate, magnification 200× (Keyence VHX-7000).

**Figure 2:**
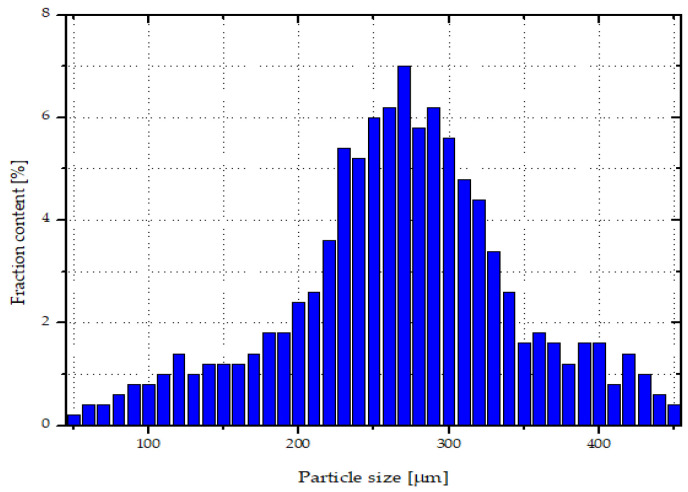
Size distribution of silica particles.

Silica waste obtained from the metallurgical industry, under the name SIMLIC (Re Alloys, Łaziska Górne, Poland) was applied as a filler (Figure 1). Silica waste was in the form of spherical agglomerates; their size ranged from ~50 to 450 µm (Figure 2). Single particle size was determined using an optical microscope - Keyence VHX-7000 (Osaka, Japan). According to the manufacturer’s specifications, silica was characterized by the following composition: silica (IV) oxide ~94 wt%, carbon (C) ~2 wt%, magnesium oxide (MgO) ~1 wt%, and other substances—mainly metal oxides such as iron (III) oxide (Fe_2_O_3_), sodium oxide (Na_2_O), and potassium oxide (K_2_O)—the amounts of which did not exceed 1 wt%.

### 2.2. Preparation of Samples

The homogenization of PP and silica was performed by means of an extrusion line for granulation, which included a single-screw extruder produced by the Institute of Plastics Processing (Metalchem, Toruń, Poland). The screw diameter (*D*) was 25 mm, and the ratio of the screw length to its diameter (*L*/*D*) was 30. In the compression zone, the screw had elements that intensified the mixing process. The temperatures of the individual zones of the plasticizing system were 135 °C (in the feeding zone), 180 °C (in the compression zone), 200 °C (in the metering zone), and 200 °C (in the head). The rotational speed of the screw was 140 rpm. Before the extrusion process, the silica powder was dried for 4 h at 110 °C using a FED 115 Binder dryer (Tuttlingen, Germany). There were six compositions prepared, each with different assumed content of silica waste in the range of 0–10 wt%. Table 1 presents a list of compositions with their signatures.

The samples for further tests were produced via injection molding using an e-victory 110 injection molding machine manufactured by Engel (Schwertberg, Austria), with a clamping force of 1100 kN, maximum injection volume of 154 cm^3^, and a screw diameter of 35 mm. The device was equipped with a two-cavity injection mold, which allowed for the production of test samples with the following dimensions: 100 mm (length), 50 mm (width), 1 mm (thickness). Finishing the surface of the molding cavity (honing No. 400) made it possible to obtain the samples with the following surface parameters: *Ra* 0.4 µm, 12 CH on the Charmilles scale (VDI 3400). All parameters of the injection molding process are presented in Table 2. Before the samples were produced, the granules were dried for 4 h at 110 °C using an FED 115 dryer (Tuttlingen, Germany).

### 2.3. Measurements of Morphology

An LEXT OLS4000 (Tokyo, Japan) 3D Measuring Laser Microscope (magnification 4284×) was used in the analysis of the silica dispersion in the polypropylene matrix after processing (extrusion of granulates and injection of samples). The specimen sections for microscopic analysis were prepared by dynamically breaking the previously frozen samples at a temperature of −40 °C.

### 2.4. Measurements of Thermal Properties

In order to assess the real silica content in the polypropylene matrix, tests were carried out using a Netzsch F1 209 Libra thermogravimeter (Selb, Germany). Samples of ~20 mg were heated under nitrogen from 25 to 600 °C at a rate of 10 °C/min. Three measurements were made for each of the samples. The mineral filler content was assumed to be the residual mass of the thermal decomposition of the samples.

The thermal properties were determined via differential scanning calorimetry (DSC) using the DSC 214 Polyma apparatus from Netzsch (Selby, Germany). Samples weighing 8–10 mg were heated to 220 °C in a nitrogen atmosphere. After two minutes of exposure at the set temperature, the samples were cooled to 20 °C and, after another two minutes, were reheated to 220 °C. The heating and cooling rate was 10 °C/min. The results of DSC tests were used to determine the degree of crystallinity (*X_C_*) using the following formula:

(1) $$X_{C}=\frac{∆H}{∆H_{C}\cdot \varphi }\cdot 100\%$$

where Δ*H_C_* is the melting enthalpy of the 100% crystalline PP, and *φ* is the weight fraction of PP matrix. On the basis of literature data [47], it was assumed that the melting enthalpy of PP with 100% crystallinity is 209 J/g.

### 2.5. Density Measurements

Density (*ρ*) testing of PP samples with different silica waste content was carried out using the immersion method. An AD 50 (Axis, Gdańsk, Poland) laboratory scale was used in these measurements. The device was equipped with a set that allowed it to determine the mass of the samples both in the air and after immersion in a liquid, which was methyl alcohol with a density (*ρ_L_*) of 0. 792 g/cm^3^. The tests were carried out at a temperature of 23 ± 2 °C. The density of individual samples was calculated using the following formula:

(2) $$\rho =\frac{m\cdot \rho_{L}}{m-m_{L}}$$

where the symbols m and *m_L_* denote the mass of the sample determined in the air and in the immersion liquid, respectively, while the symbol *ρ_L_* denotes the density of the immersion liquid

### 2.6. Measurements of Tensile Properties

The mechanical properties of the samples during the static tensile test were determined using a ZwickRoell Z030 universal testing machine (Ulm, Germany). The device was equipped with a measurement head with a nominal value of up to 30 kN. The extension rate during the measurements of elastic modulus was 1 mm/min, and the tensile speed was increased to 50 mm/min until the specimen broke. Samples with a dog-bone shape were cut out from the previously produced moldings using a specially prepared die. Specimens prepared in this way had the following dimensions: 1 mm (thickness), 10 mm (width at the narrow part), 40 mm (length of the narrow parallel-sided section), and 95 mm (overall length). Due to the geometry of the samples (i.e., thickness, measuring distance), a mechanical extensometer was not used in the tests. The tests were carried out at a temperature of 23 °C for 10 samples.

### 2.7. Laser Marking

**Figure 3:**
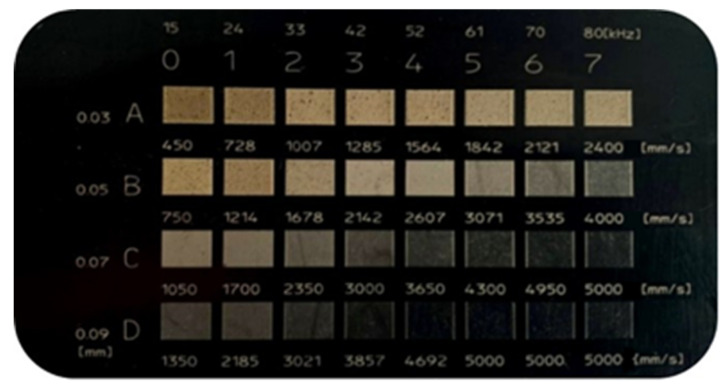
An exemplary matrix of marking of individual graphic fields for specific parameters of the laser beam (S7). (**A**–**D**)—width of the path (from 0.03 to 0.09 mm); (**0**–**7**)—frequency of the beam pulses (from 15 to 80 kHz); speed (from 450 to 5000 mm/s).

Samples with different silica content were modified via laser radiation at the wavelength *λ* = 1064 nm. A TruMark Station 100 Nd:YAG laser marking machine (Trumpf Group, Ditzingen, Germany) was used for surface treatment. In preliminary tests, the samples were marked with a matrix of graphic fields made with various applied parameters of the laser beam. The changeable parameters of the laser beam were the head linear speed (from 450 to 5000 mm/s), the width of the path (from 0.03 to 0.09 mm), and the frequency of the beam pulses (from 15 to 80 kHz). An example of a specimen showing marked graphic fields obtained with the use of variable parameters is presented in Figure 3.

Further modifications of the surface area were performed with the following laser operating conditions: constant frequency of laser pulses (15 kHz), variable speed of the laser beam (450–1350 mm/s), and variable path width (0.03–0.09 mm). These parameters resulted in the most favorable visual effects of surface marking, obtained at the stage of preliminary tests (contrast of the marking area in relation to the background and a wide range of obtained colors). A detailed list of laser marking conditions for individual fields with signatures is presented in Table 3. The marked graphic fields had the shape of a square with a side length equal to 16 mm.

### 2.8. Spectrophotometric Color Analysis

The differences in color between the marked surfaces and the background were determined via colorimetric tests using a Ci62 colorimeter (X-Rite, Grand Rapids, MI, USA). To analyze the results, the CIELAB color space was used, within which the parameters L* (brightness, values from 0—black to 100—white), a* (color change from green to red), and b* (color change from yellow to blue) were determined for each measurement area. Measurements were performed on laser-marked surfaces and surfaces of samples before marking (background), by making 5 measurements on each surface. The color difference between the surface of the sample before marking and the marked surface was assessed by analyzing the value of the total color deviation (Δ*E*). This parameter was calculated using the following formula:

(3) $$∆E=\sqrt{{\left(\Delta L^{*}\right)}^{2}+{\left(\Delta a^{*}\right)}^{2}+{\left(\Delta b^{*}\right)}^{2}}$$

### 2.9. Optical Surface Analysis

Optical analysis of the marked graphic fields was performed using a Keyence VHX-7000 digital microscope (Osaka, Japan), equipped with a universal VH-Z100R (Osaka, Japan) zoom lens with a magnification of 100×–1000×. In addition to microscopic observations, based on the image analysis of photographs made with the depth composition, the degree of filling in the obtained graphic characters was determined by calculating the sum of laser beam interference areas on individual marked signs. Moreover, roughness measurements were carried out involving the determination of the arithmetic mean deviation of the assessed profile (*Ra*). This parameter value was determined on the basis of averaging 10 test sections (5 on the vertical axis, 5 on the horizontal axis). The degree of filling of the marked area, along with roughness, was determined for a magnification of 500×, at which the dimensions of the tested surfaces were equal to 600 µm × 450 µm.

**Figure 4:**
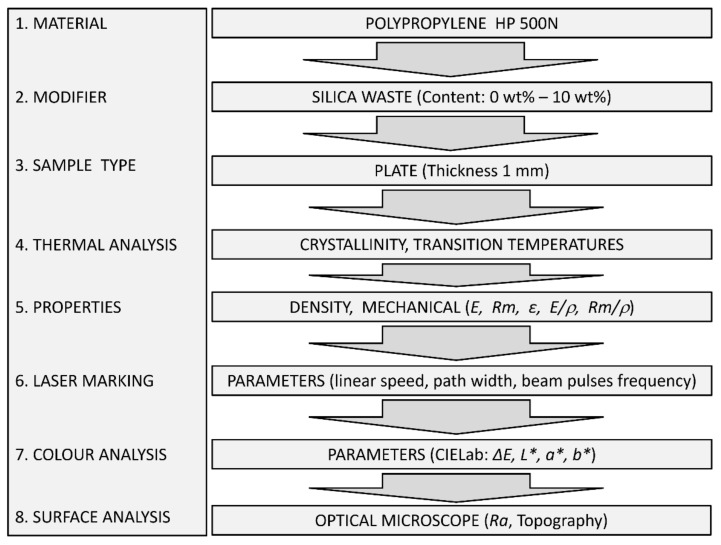
Experiment flow diagram with input and output data.

A diagram explaining the individual stages of the experiment is shown in Figure 4.

## 3. Results

### 3.1. Morphology of the Samples

**Figure 5:**
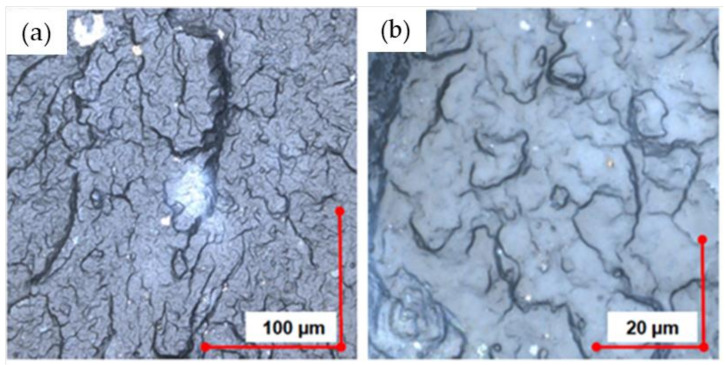

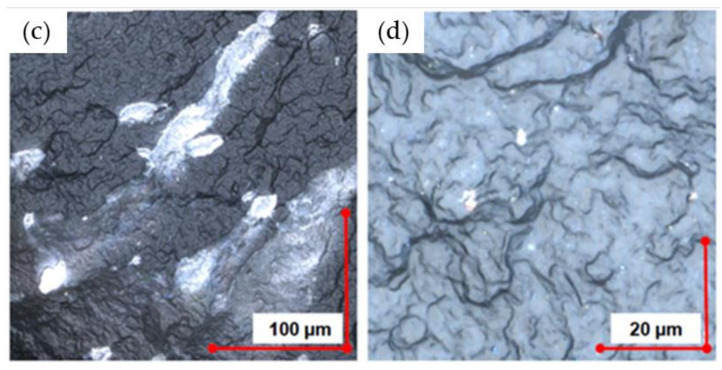
Break section of PP samples (magnification 4284×) filled with waste silica: (**a**,**b**) 3% content; (**c**,**d**) 10% content.

Based on the analysis of the fracture morphology of the PP/silica samples, it was found that the filler agglomerates remained ground in plasticizing systems—both extruders and injection molding machines. Inorganic filler particles (white irregular particles) ranged from <1 to ~100 µm. Sample photos depicting the dispersion of silica particles in the polymer matrix are shown in Figure 5.

### 3.2. Thermal Properties

**Figure 6:**
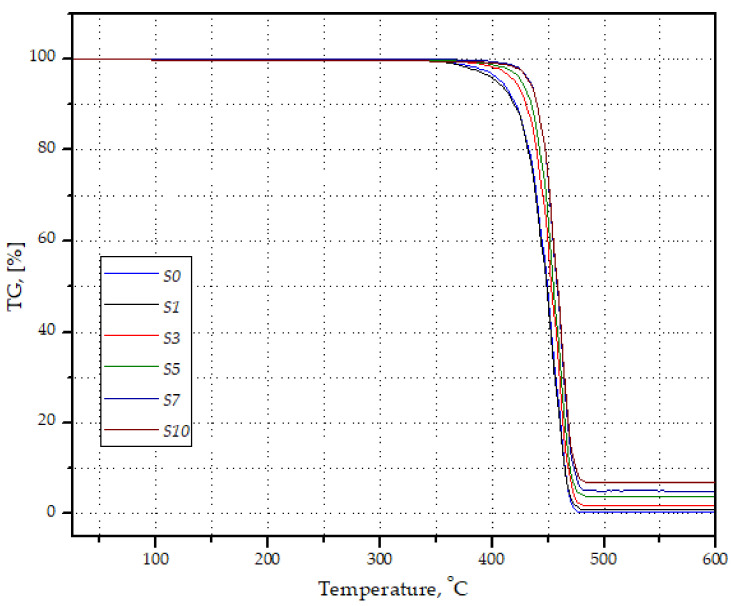
Selected curves of weight loss of PP/silica samples as a function of temperature, recorded during TG tests.

Based on the thermogravimetric tests, it was found that the real filler content differed from the assumed content. After the decomposition of the polymer matrix in the case of sample S1, an average of ~0.7 wt% mineral filler was left. For sample S10, where the assumed silica content should be 10 wt%, the average filler content was equal to 7.67 wt% (Table 4). Exemplary curves of the mass loss of samples as a function of temperature are presented in Figure 6. Therefore, it can be assumed that despite the precise dosing of the components, the filler particles could settle in the plasticizing system of both the extruder and the injection molding machine, which resulted in a lower proportion of silica in the material than assumed. In the following sections of this article, the real rounded value of silica in the PP matrix is used to describe the test results.

**Figure 7:**
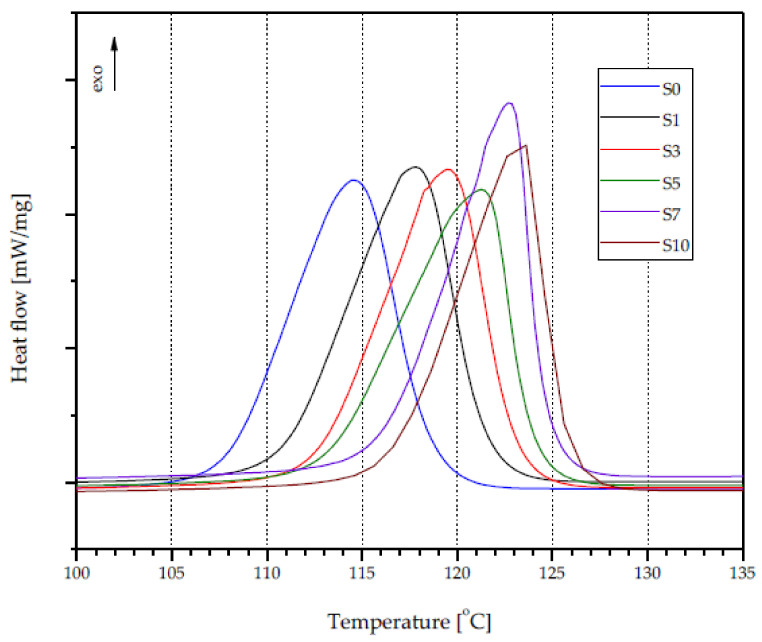
DSC crystallization curves of PP silica composites.

The introduction of silica waste into the polypropylene matrix resulted in a gradual increase in the crystallization temperature (*T_c_*) of the obtained composites, as shown in Figure 7. The *T_c_* value for unfilled PP was 114.5 °C, while the values recorded for compositions S5 and S10 were equal to 121.6 °C and 123.3 °C, respectively (Table 5), indicating a nucleation role of the silica. The registered maximum increase in *T_c_* (8.8 °C) was lower than the result (16 °C) obtained by Romankiewicz et al. [48] using 1,3:24-bis (3,4-dimethylobenzylidene) sorbitol (DMDBS) as a nucleating agent. Nevertheless, the higher crystallization temperature of PP in composites with silica waste offers advantages for application purposes, particularly by reducing the cooling time during injection molding processing. However, the presence of silica in the PP may result in an increase in the viscosity of the plasticized material, which may make it difficult to fill the injection mold cavity. The presence of silica in the PP did not significantly affect the melting point (*T_m_*) of the thermoplastic matrix of the composites, the recorded values of which were around 4 °C (Table 5). The *T_m_* of unfilled PP was equal to 166.3 °C. In the case of the obtained composite materials, PP melting at temperatures from 166.3 °C (S1) to 170.7 °C (S7) was observed. Moreover, an introduction of ~0.7 wt% silica (S1) caused a slight increase in the melting enthalpy (Δ*H*) of PP (88.57 J/g) compared to unfilled PP (88.48 J/g), despite the decrease in the amount of thermoplastic polymer in the total mass of the composite sample. However, a further increase in the proportion of filler in the material resulted in a decrease in Δ*H*. For the S10 composition, the recorded value was equal to 87.36 J/g.

The degree of crystallinity of unfilled PP reached a value of 42.3% (Table 5). With the increase in the amount of silica in the composites, the Xc of polypropylene increased, reaching the highest value of ~45%. This effect was observed for the samples with the highest degree of filling (S7 and S10).

### 3.3. Density and Tensile Properties

With the increase in the content of silica waste in the PP matrix, a gradual, linear increase in the density of the obtained composites was recorded (Table 6). A density of 0.906 g/cm^3^ was found for the unfilled PP. In the case of 1% filler content, the material density increased to 0.91 g/cm^3^, i.e., by 0.44%. For composition S5, a 2% increase in density (0.924 g/cm^3^) was recorded. The composition with the highest tested degree of filler (S10) was characterized by a density of 0.95 g/cm^3^. The higher density of composites compared to unfilled PP is unfavorable for application purposes; its consequence may be an increase in the weight of moldings made of this type of material.

The introduction of silica waste into the polypropylene matrix resulted in an increase in the modulus of elasticity (*E*), as determined during the static tensile test. The unfilled PP was characterized by an *E* value equal to 1460 MPa (Table 6). The introduction of 0.7 wt% of filler to the matrix resulted in a nearly 4% increase in the modulus of elasticity (1516 MPa). Samples with 10% silica content were characterized by a 20% increase in *E* value compared to pure PP. Moreover, small amounts of filler dispersed in the PP matrix resulted in an increase in tensile strength at yield. The *σ_y_* value of composition S1 was approximately 3% higher (35.9 MPa) than that of unfilled PP (34.9 MPa). The highest *σ_y_* value (36.3 MPa) was recorded for compositions S7 and S10. It can be assumed that a further increase in the degree of filling of the composites may result in a decrease in the tensile strength of the material at yield. Based on previous tests [9], it was found that in the case of samples with a thickness of 4 mm, the addition of 20 wt% silica results in a reduction of 15% in the tensile strength at yield compared to the unfilled matrix. This may be due to the difference in the wall thickness of the moldings, which in the current tests was four times smaller. Moreover, it can be concluded that the changes in the mechanical properties (improvement of *σ_y_*) of the obtained composites, recorded with the increase in the content of silica, may be the result of the interaction between the matrix and the filler.

Along with the increase in the content of microsilica, a decrease in the value of elongation at the yield point was observed. In the case of unfilled PP, the value of elongation at yield was equal to 9%, while for the composite with highest filler content, a value of 6% was recorded. The increase in stiffness was caused by the influence of the filler on the fraction size (predominant particle size below 1 µm—tube 5). Similar relationships between the values of the parameters of mechanical properties and the degree of filling of the PP matrix with microsilica were noticed by Pustak et al. [49]. However, the results of elongation at yield described in this paper are more favorable. In the case of the S10 sample, the elongation at yield value was less than 19% lower compared to the results of Pustak et al., where this value dropped by over 53% (with 8% silica filling). This is a very important parameter, because elongation at yield point is a threshold parameter in the design and testing of polymer parts.

**Figure 8:**
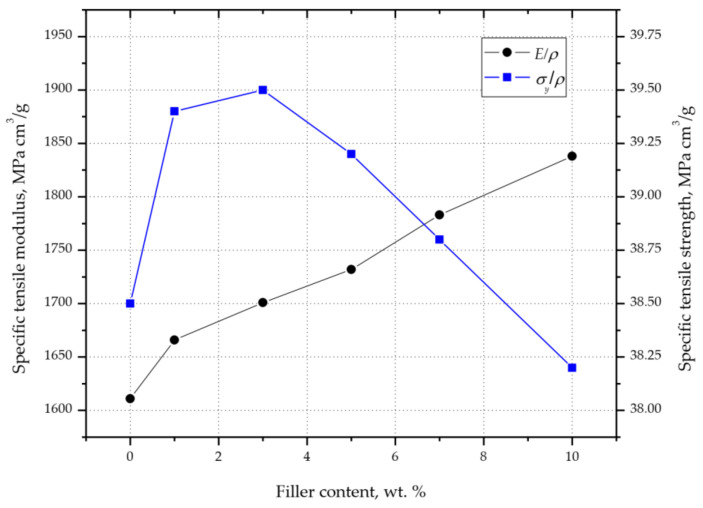
Changes in PP/silica composites’ mechanical properties as a function of filler content.

Based on the results of the density, modulus of elasticity, and tensile strength tests, the specific modulus of elasticity (*E*/*ρ*) and the specific tensile strength (*σ_y_*/*ρ*) were determined (Figure 8). Despite the increase in density, the *E*/*ρ* value increased gradually with the increase in the filler content from 1611 (in the case of unfilled PP) to 1838 MPa cm^3^/g (S10 composition). A different course of changes in the values was observed for the *σ_y_*/*ρ* value. After initially increasing from 38.5 MPa cm^3^/g (PP), and reaching a maximum value of 39.5 MPa cm^3^/g for composition S3, the *σ_y_*/*ρ* value began to decrease. This was probably due to the stabilization of the tensile strength values (samples S3–S10) and the constant increase in the density of the composites with the increase in the degree of filling (Table 6). Even though the tensile strength of composition S10 was higher than that of unfilled PP, *σ_y_*/*ρ* reached a lower value; therefore, it should be concluded that for fillings up to 5.1 wt%, the higher *σm* value of the composite compensates for the increase in its density.

In summary, the absence of any deterioration of the tensile strength due to the dosing of waste silica—and even improvement of this parameter in the range from 0.7 to 1.5 wt%—justifies the implementation of further research in the field of laser marking. The determined mechanical properties of PP composites with silica should not significantly reduce the application potential of this silica as an LMA.

### 3.4. Color Changes

The color analysis of the moldings before the marking process revealed that the presence of silica in the PP matrix caused the color change of the samples from semi-transparent (natural color of PP) to dark. This effect was obtained mainly due to the presence of carbon in the powder filler, which commonly acts as a black dye. Similar observations in the case of aged PVC modified with nanosilica were recently published by Tomaszewska et al. [16], wherein a gradual change in color (lowering the L* parameter value by ~50%) was achieved by extending the mixing of the sample melt (rolling time). In the tests carried out with waste silica (with the value of the L* parameter similar to the value obtained in the study by Tomaszewska et al.), a more intense change in the L* parameter was obtained (change of 71% to the initial value). This change was most likely due to the presence of sufficient carbon dopants in the waste filler to color the polymer.

**Figure 9:**
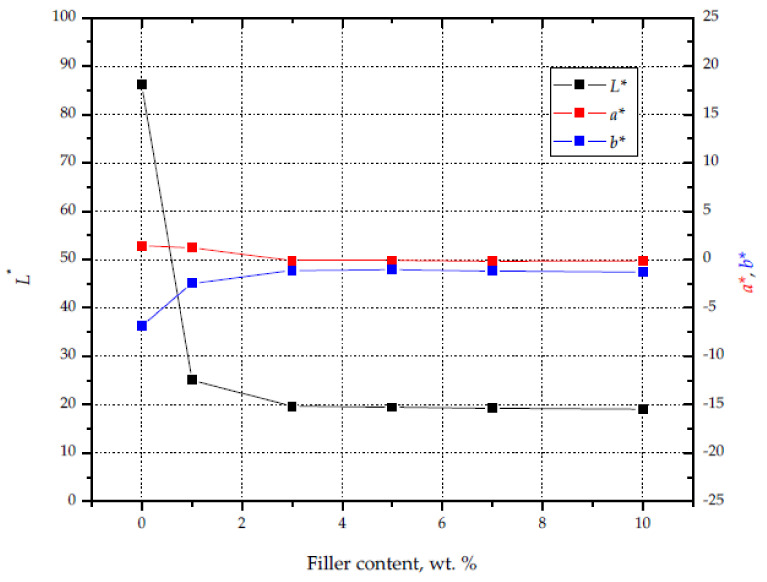
Influence of silica content on L*, a*, and b* values.

The increase in the proportion of silica in the matrix resulted in an increase in the intensity of the black color of the material. The observed changes were parameterized using the L*, a*, and b* parameters (Figure 9). The color change had the greatest impact on the value of the L* parameter (brightness), which in the case of unfilled PP reached 86. The introduction of 0.7 wt% filler caused a decrease in the L* parameter to 25, while for composition S10 a value of 19 was recorded. The decrease in the value of the described parameter resulted in a darker color of the moldings. The largest changes in the values of the L*, a*, and b* parameters were recorded for samples S0–S3. In this range of the degree of filling, the value of a* decreased from 1.44 to 0.09, while the value of b* decreased from 6.87 to 1.13. A further increase in the content of silica waste did not change the described parameters, indicating a stabilization of color starting from 1.5 wt% silica content.

**Figure 10:**
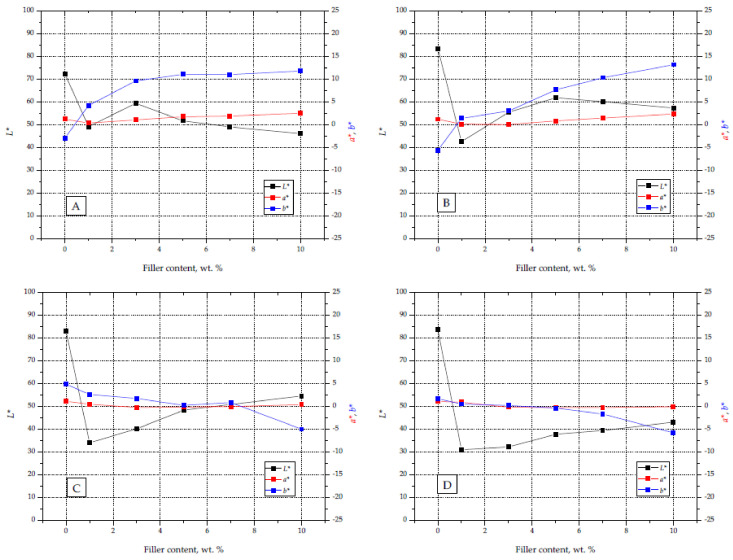
Charts with L*, a*, and b* parameters for graphic signs made on samples with different filler content and different laser beam parameters (**A**–**D**).

The introduction of silica waste to the PP matrix, in addition to the black color of the moldings, made it possible to effectively apply graphic signs on their surface. The color of the obtained graphic fields depended on the laser operating parameters used and the amount of filler in the material. Figure 10 shows the course of changes in the values of the L*, a*, and b* parameters for the marked surfaces, obtained with different laser operating parameters and different contents of silica waste. In the case of attempts to mark the moldings without the addition of silica, no clear changes on the surface were obtained. This fact is confirmed by the high L* values of the surfaces exposed to the laser beam, which were similar to those recorded for the background. This effect justifies the need for research in the area of LMA selection for marking polymers from the polyolefin group. The L* index values obtained by the B parameters (L* 55–60) are similar to those obtained by Czyżewski et al. [29]. An industrial LMA compound and a black dye were used for these studies, obtaining L* parameter values at the level of 40–60.

The change in the observed color of the marked surfaces was most noticeable in the analysis of changes in the L* parameter (brightness), the values of which changed to the greatest extent, from ~30 (S1 D) to 60 (S5 B). Moreover, the color change was also indicated by an increase in the b* component value (in the case of A and B laser operating parameters) and a decrease in the value of this component for the remaining marking conditions.

**Figure 11:**
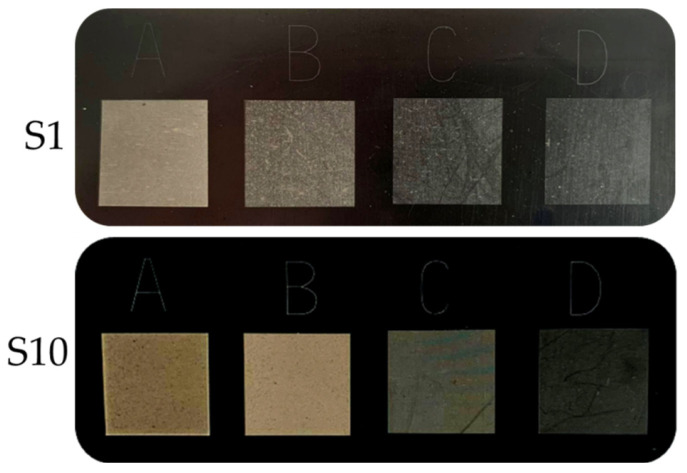
Graphic signs applied with different laser operating parameters: (**A**) 450 mm/s, 0.03 mm; (**B**) 750 mm/s, 0.05 mm; (**C**) 1050 mm/s, 0.07 mm; (**D**) 1350 mm/s, 0.09 for samples S1 and S10.

The addition of silica caused a slight shift in the a* value for the graphic signs towards a red color, while in the case of b* there was a shift towards a yellow color. As a result, taking into account all of the described parameters, the images of marked fields were characterized by an increased contrast between the graphic symbol and the background surface. On the moldings with a higher content of silica and laser parameters A and B, graphic signs in shades of yellow and brown were obtained, while samples with lower filler content and marking parameters C and D were characterized by shades of gray. Examples of moldings with applied graphic signs made with different laser operating parameters are presented in Figure 11.

**Figure 12:**
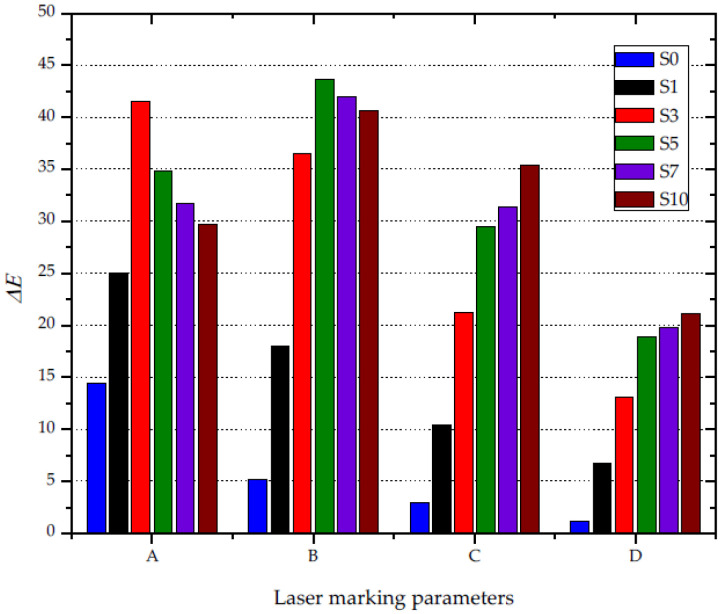
The impact of the content of silica waste and the laser operating parameters on the Δ*E* parameter.

The analysis of the Δ*E* value (Figure 12)—i.e., the color difference between the background and the obtained graphic symbol—shows that the highest contrast was obtained using low values of the laser head work speed (450–750 mm/s), combined with a large beam focus (0.03–0.05 mm) and low pulse frequency (15 kHz). The increase in laser head velocity and path width resulted in lower contrast. The highest value of Δ*E* (44) was recorded for the operating parameters of laser B, while the highest value of Δ*E* for parameters C and D did not exceed 35. Moreover, it was found that the graphic characters with the greatest contrast against the black background were obtained for compositions containing 1.5 wt% (A) and 3.5 wt% (B) silica waste. Such a composition of silica and PP, along with the described laser parameters, had the greatest impact on the susceptibility of the material to the absorption of laser radiation. Increasing the concentration of silica above 3.5 wt% has little effect on the change in the contrast between the graphic symbol and the background for parameters A and B. Czyżewski et al. [50] obtained lower contrast between the marked surface and the black background (Δ*E*) using a composition of polypropylene, black dye, and a commercial LMA additive in their research, whereas silica is a waste material that can simultaneously act as a dye and a marking additive.

### 3.5. Laser Surface Modification

**Figure 13:**
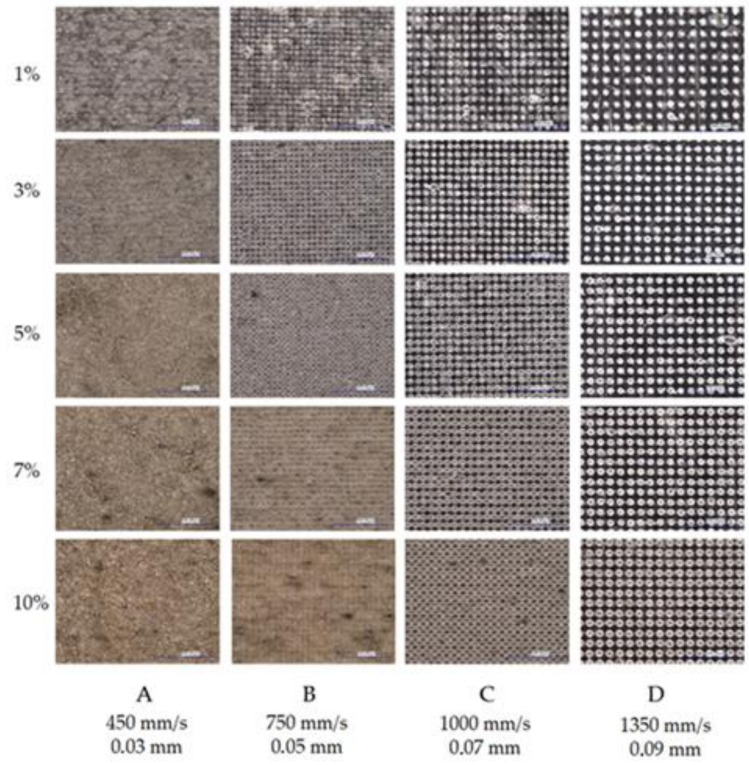
Microscopic photographs of graphic signs obtained with different parameters of the laser beam on specimens with different silica contents; magnification 200×; dimensions of investigated images 1000 µm × 1500 µm, (Keyence VHX-7000). (**A**) 450 mm/s, 0.03 mm, (**B**) 750 mm/s, 0.05 mm, (**C**) 1000 mm/s, 0.07 mm, (**D**) 1350 mm/s, 0.09 mm.

The marking of PP moldings containing silica waste as an LMA was the result of modifying the internal structure of the composite. When observing graphic signs, using an optical digital microscope, it is possible to note an increase in the intensity of surface modification by the laser beam, along with an increase in the filler content in the PP matrix (Figure 13). This was confirmed by the larger size of individual points in the places of interference of the laser beam (in the case of compositions with a high percentage of the modifier). This effect was clearly visible for the markings using parameters C and D. Moreover, the increase in the intensity of the graphic signs was recorded at a low laser head speed (450–750 mm/s), a strong focus of the beam (0.03–0.05 mm), and low pulse repetition frequency (15 kHz). For the parameters selected in this way, the marked area was modified several times by successive passages of the laser beam and the overlapping of surface fragments. Local blackening on the marked surface of the material may result from the uneven distribution of silica in the PP matrix.

**Figure 14:**
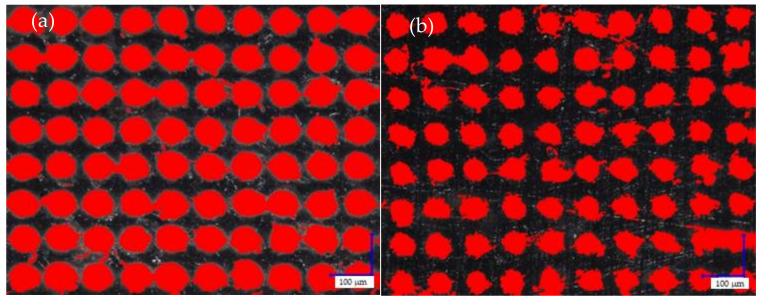
The degree of filling of the graphic fields with the interference of the laser beam, caused by thermal defects: (**a**) 0.7 wt% silica 1350 mm/s, 0.09 mm; (**b**) 7.7 wt% silica 1350 mm/s, 0.09 mm; magnification: 400×; dimensions of investigated images: 750 µm × 1000 µm, (Keyence VHX-7000).

The size of individual points of laser interference on the marked surface had a significant impact on the degree of filling of the graphic field with heat distribution points (Figure 14). The increase in filler content in the PP matrix resulted in an increase in laser radiation absorption, causing an increase in the degree of filling of the graphic symbol (Table 7). For example, for the marking parameter B, the filling degree changed from 64% (S1) to 93% (S10). Higher laser radiation energy (A) caused an increase in the intensity of surface modification. In the case of using the lowest laser speed and the smallest path width, regardless of the amount of filler in the material, the degree of filling in the graphic field was equal to 100%.

It was found that the degree of filling in the graphic field significantly determined the color that was observed on the marked surface. However, the highest contrast was obtained for sample S5, with the operating parameters of laser B, i.e., 85% of the filling degree of the graphic symbol. Therefore it may be concluded that the overlapping of individual points of interference of the laser beam does not always lead to the achievement of the greatest possible contrast between the graphic symbol and the background.

**Figure 15:**
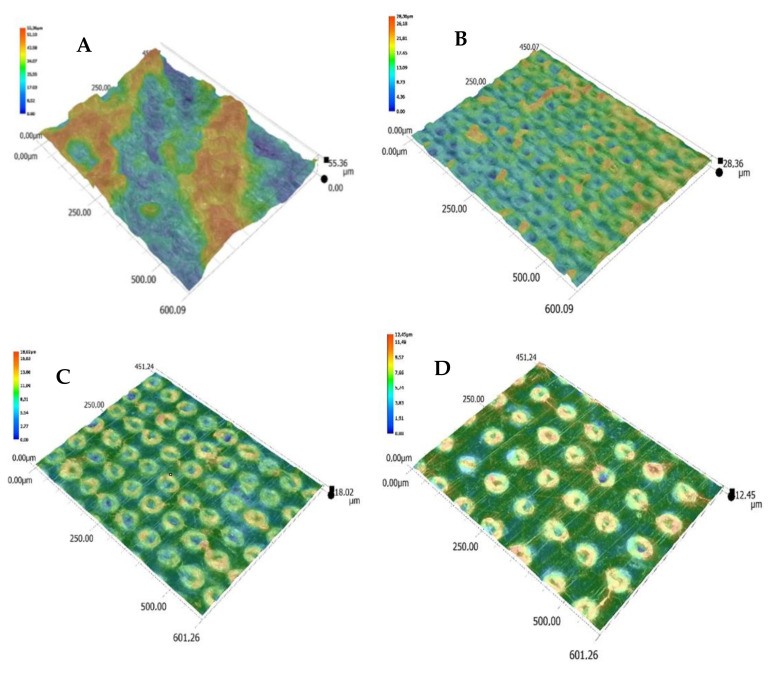
Change in the molding surface (S5) topography as a result of the laser beam action with various parameters: (**A**) 450 mm/s, 0.03 mm; (**B**) 750 mm/s, 0.05 mm; (**C**) 1050 mm/s, 0.07 mm; (**D**) 1350 mm/s, 0.09; magnification: 500×; dimensions of investigated images: 450 µm × 600 µm (Keyence VHX-7000).

Moreover, advanced image analysis of microscopic photographs of the obtained graphic marks indicates that one of the effects of the laser beam on the surface of the moldings was the foaming process. The laser beam caused local thermal degradation of the composition’s components, and the resulting small gas bubbles were enclosed under a thin layer of polymer. This was confirmed by 3D images of the surface of the moldings after the marking process (Figure 15). The effect of the growing intensity of electromagnetic radiation was an increase in the height of the foamed areas above the surface of the moldings.

**Figure 16:**
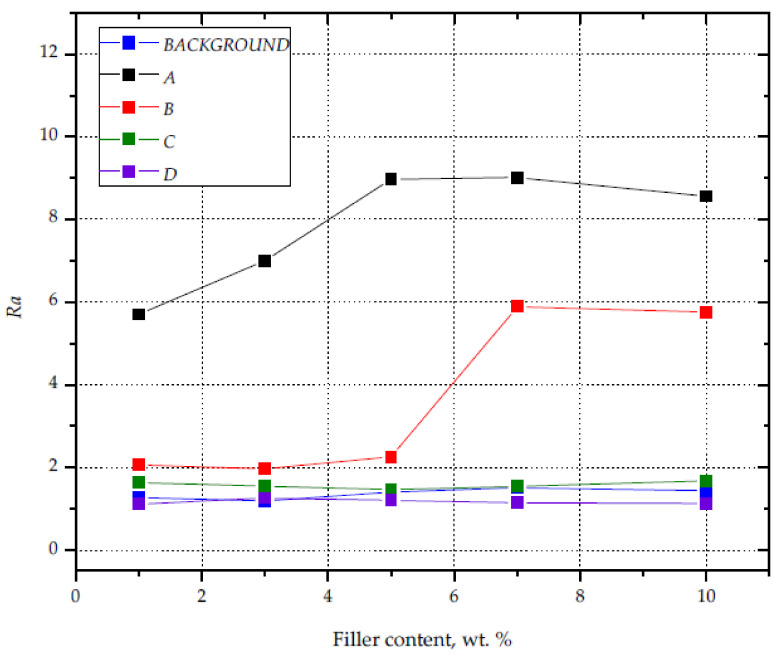
Surface roughness of samples with varying silica contents and different laser beam parameters.

The result that confirms the intensity of modification of the surfaces subjected to the laser marking process is the roughness of the obtained graphic marks. The graphic marks obtained with the laser A performance parameters were characterized by the highest roughness, regardless of the silica content in PP (Figure 16). The *Ra* value varied from 5.70 (S1) to 9.01 for the composition with 5.1 wt% filler content. High roughness (*Ra* = 6) was also recorded for the samples with the highest contents of silica (S7 and S10), the surface of which was marked with the use of the laser B parameters. It should be noted that high *Ra* values are correlated with the degree of filling in the graphic characters (higher than 90%), which takes place when the individual points of interference of the laser beam overlap, resulting in a greater intensity of foaming. Marking the surface using parameters C and D did not result in a significant increase in roughness, with values similar to those recorded for the black background surface.

## 4. Conclusions

The advantage of the use of silica-based modifiers in order to apply graphic symbols on the surface of PP moldings via laser radiation was confirmed. The powdered silica waste used in the research allowed for effective dyeing of PP to black at 1.5 wt%. This was due to the presence of carbon in the waste filler used. Moreover, the applied modifier allowed us to enhance the surface of the PP moldings during the laser marking process. The greatest contrast between the graphic symbol and the background (the color of the molding) was obtained on the surface of the samples with silica contents equal to 1.5 and 3.5 wt%, simultaneously using low marking speeds, high concentration of the laser beam, and a low frequency of laser pulses. The color obtained on the surface of the laser-marked graphic signs also depends on the degree of filling of the field with the laser beam interference points, as well as the roughness of the surface. The increase in the roughness of the marked surfaces is correlated with the intensive foaming process in the areas of electromagnetic radiation accumulation.

In addition, dosing the silica waste into the polypropylene matrix at proportions from 0.7 to 7.7 wt% resulted in an increase in the crystallization temperature, which may have a positive effect on the shortening of the cycle time of the injection process of composite moldings. As in the case of other mineral fillers, increases in density and Young’s modulus were observed, proving the better stiffness of the obtained composites. In summary, it can be said that silica waste can act as a filler that enhances mechanical properties, as a heterogeneous nucleating agent, as a dye, or as an additive to facilitates the laser marking process.

## Figures and Tables

**Table 1:** Signatures of individual mixtures of polypropylene with silica waste.

**Signature**	S0	S1	S3	S5	S7	S10
**Assumed silica content, wt%**	0	1	3	5	7	10

**Table 2:** Parameters of the injection process of PP samples with silica.

Processing Parameters	Value
Feed zone temperature	210 °C
Transition zone temperature	220 °C
Metering zone temperature	230 °C
Nozzle temperature	230 °C
Mold temperature (°C)	50
Holding time (s)	5
Cooling time (s)	5
Injection speed (cm^3^/s)	70
Holding pressure (MPa)	60

**Table 3:** The laser beam parameters for specific graphic fields used during the laser marking process of the actual research samples.

Process Parameter	Value			
Pulse frequency (kHz)	15			
Head velocity (mm/s)	450	750	1050	1350
Diameter of single impulse (mm)	0.03	0.05	0.07	0.09
Description of marked area	A	B	C	D

**Table 4:** Results of TG measurements.

**Signature**	S0	S1	S3	S5	S7	S10
**Residual mass, (wt%)**	0.09	0.73	1.45	3.52	5.13	7.67

**Table 5:** Thermal properties of PP/silica composites.

Sample	Crystallization Temperature (°C)	Melting Temperature (°C)	Melting Enthalpy (J/g)	Crystallinity (%)
S0	114.5	166.3	88.48	42.3
S1	117.8	166.6	88.57	42.7
S3	119.5	168.4	89.66	43.5
S5	121.6	168.9	89.98	44.6
S7	122.8	170.7	88.79	44.8
S10	123.3	168.9	87.36	45.3

**Table 6:** Density and mechanical properties of PP/microsilica composites.

Material	Density (g/cm^3^)	Tensile Modulus (MPa)	Tensile Strength at Yield (*σ_y_*), (MPa)	Elongation at Yield (%)
S0	0.906 ± 0.0012	1460 ± 12.6	34.9 ± 0.05	8.90 ± 0.11
S1	0.910 ± 0.0009	1516 ± 10.4	35.9 ± 0.09	8.55 ± 0.09
S3	0.914 ± 0.0009	1555 ± 15.0	36.1 ± 0.08	8.05 ± 0.05
S5	0.924 ± 0.0011	1601 ± 19.3	36.2 ± 0.11	7.99 ± 0.04
S7	0.935 ± 0.0016	1667 ± 13.6	36.3 ± 0.09	6.62 ± 0.08
S10	0.950 ± 0.0012	1746 ± 11.8	36.3 ± 0.07	6.35 ± 0.04

**Table 7:** The percentage degree of filling of the graphic symbols with the laser beam interference points.

Sample	Filled Area (%)			
	A	B	C	D
S1	100	64	50	32
S3	100	75	54	42
S5	100	85	68	45
S7	100	91	77	51
S10	100	93	81	60

## Data Availability

The data presented in this study are available on request from the corresponding author.
